# Psychological distress, distress tolerance, and cannabis-related problems among university students in Türkiye: a mixed-methods study

**DOI:** 10.3389/fpubh.2026.1799686

**Published:** 2026-06-25

**Authors:** Metin Çınaroǧlu, Eda Yılmazer

**Affiliations:** 1 Psychology Department, Faculty of Administrative and Social Science, Ìstanbul Nişantaşi University, Ìstanbul, Türkiye; 2 Psychology Department, Faculty of Social Science, Beykoz University, Ìstanbul, Türkiye

**Keywords:** cannabis use, distress tolerance, emotion regulation, mixed-methods, psychological distress, university students

## Abstract

**Background:**

Cannabis use among university students has been increasingly linked to psychological distress; however, most evidence comes from Western contexts, and little is known about how cannabis use intersects with emotional functioning in non-Western settings. In Türkiye, where cannabis use remains illegal and highly stigmatized, students who use cannabis may represent a particularly vulnerable group. This study examined the associations between cannabis-related problems, psychological distress, and distress tolerance among Turkish university students, and explored students' lived psychological experiences of cannabis use.

**Methods:**

A sequential explanatory mixed-methods design was employed. In the quantitative phase, 512 university students from Istanbul, Ankara, and Izmir completed self-report measures assessing cannabis-related problems (Cannabis Use Problems Identification Test), depressive symptoms (Beck Depression Inventory–II), anxiety symptoms (Beck Anxiety Inventory), and distress tolerance (Distress Tolerance Scale). In the qualitative phase, semi-structured interviews were conducted with a purposively selected subsample of 43 students with elevated cannabis-related problems and/or psychological distress. Quantitative data were analyzed using descriptive and correlational analyses, while qualitative data were examined using reflexive thematic analysis. Findings were integrated at the interpretation level.

**Results:**

Greater cannabis-related problem severity was moderately associated with higher levels of depressive and anxiety symptoms and with lower distress tolerance. Qualitative analyses yielded four overarching themes: (1) psychological effects of cannabis use, (2) cannabis use as a distress regulation strategy, (3) perceived loss of control and ambivalence, and (4) academic and interpersonal consequences. Integration of quantitative and qualitative findings suggested a cyclical pattern in which psychological distress motivates cannabis use for short-term relief, which in turn undermines distress tolerance and contributes to further psychological and functional difficulties.

**Conclusions:**

Cannabis use among Turkish university students appears closely intertwined with emotional distress and reduced capacity to tolerate negative affect. Rather than being solely recreational, cannabis use often functions as a maladaptive coping strategy. Interventions targeting emotion regulation and distress tolerance may be particularly beneficial in this population. These findings highlight the importance of integrated mental health and substance-use approaches within university settings, especially in culturally conservative contexts.

## Introduction

Cannabis is one of the most widely used psychoactive substances among young adults worldwide, including university student populations (1). In Western countries, nearly half of emerging adults report recent cannabis use [e.g. 42.6% past-year prevalence in U.S. college-aged samples (2)]. In contrast, cannabis use remains relatively less common in more conservative, non-Western contexts; in Türkiye, studies have found lifetime cannabis use rates of only around 2%−12% among university students (3). This discrepancy reflects not only differences in availability and legal status, but also cultural attitudes—a strong societal stigma surrounds any drug use in Türkiye, and cannabis is far less normalized than in many Western settings. Those who do use cannabis in such contexts may therefore represent a more hidden or vulnerable minority, underscoring the importance of understanding their experiences. The present study focuses on *emerging adulthood*—roughly ages 18–25—a developmental stage when individuals often enter university, take on new social roles, and experience heightened exploration and instability. Arnett's theory of emerging adulthood posits that this life stage is marked by identity exploration and novelty-seeking, which can include risk behaviors like experimenting with substances (4). Indeed, university students, in late adolescence/early adulthood, face unique psychosocial challenges: accelerated biological changes, emotional instability, academic pressures, and social transitions can make them more prone to mood fluctuations and risk-taking (5). University life often entails increased freedom and peer influence, which may facilitate substance use (6). However, in societies where drug use is highly taboo, these behaviors also carry potential for conflict and internalized guilt (7). It is within this *university context in Türkiye*—characterized by both the developmental propensity for experimentation and a cultural climate of disapproval—that we situate our investigation of cannabis use and psychological wellbeing.

To ground this study, we draw on several interrelated theoretical frameworks explaining why young people may turn to substances like cannabis in the face of emotional distress. *Emotion Regulation Theory* (8) emphasizes the processes by which individuals influence their own emotional states. Difficulty in emotion regulation—sometimes termed *emotion dysregulation*—has been linked to substance use as a maladaptive coping strategy (9). When healthy emotion-regulation skills are lacking, a person might use an external agent (such as a drug) to modulate feelings. In fact, research has shown that individuals with greater emotion regulation difficulties are more prone to using substances for relief, and higher emotion dysregulation is associated with more severe cannabis use problems (10). This aligns with classic negative reinforcement models of addiction, in which substance use is reinforced because it provides an escape from negative affect (56). In a related vein, the *Self-Medication Theory* of Khantzian (11, 12) explicitly posits that some individuals develop patterns of substance use as a compensatory attempt to *medicate* psychological suffering. According to this theory, there is an affinity between a person's specific emotional or psychiatric struggles and the psychotropic effects of their drug of choice. In the case of cannabis, many young people report using it to calm anxiety, lift low mood, or alleviate stress—effectively treating their emotional symptoms in the short term (13, 14). This behavior conforms to an operant conditioning paradigm of negative reinforcement: if consuming cannabis reliably reduces unpleasant feelings (anxiety, sadness, etc.), the individual is likely to continue that behavior whenever distress arises (15). Over time, this *self-medication cycle* can contribute to heavier and more habitual use (16). Notably, prior studies have found that young adults who use cannabis to cope with mental health symptoms tend to exhibit *greater* problems: they have higher use frequency, more cannabis-related adverse consequences, and elevated psychological distress relative to those who use for other motives (17–19). For example, one survey of high-frequency young adult users found that 76% endorsed using cannabis to reduce issues like anxiety, depression, or insomnia, and those who did so exhibited higher risk of cannabis use disorder (20). Self-medicating with cannabis has been linked to higher rates of conditions such as generalized anxiety and panic disorder (21). Moreover, cannabis use in young adulthood is often correlated with depressive symptoms, though the causal direction is debated (22). Some longitudinal evidence suggests that early frequent cannabis use can precede and increase the risk of later depression (23), while other research indicates that pre-existing depression can drive individuals to use cannabis more frequently (24)– likely both processes occur in a reciprocal manner. This bidirectional interplay exemplifies why an integrative theoretical lens is needed: it is not simply that “cannabis causes depression” or “depression leads to cannabis,” but rather that individuals might enter a feedback loop of using cannabis to cope, which in turn may worsen mood or life problems, reinforcing further use.

A key construct linking these frameworks is *distress tolerance*, or one's capacity to withstand negative emotional states (25). Distress tolerance is considered a transdiagnostic vulnerability factor—low tolerance for distress can underlie various maladaptive behaviors, including substance use (26). Someone with poor distress tolerance tends to feel overwhelmed by emotional pain or stress and urgently seeks relief. Cannabis, with its acute anxiolytic and mood-altering effects, can be an appealing quick fix for such individuals (27). Indeed, empirical studies have identified low distress tolerance as a significant predictor of cannabis use coping motives and related problems (28). For instance, Buckner and colleagues (29) found that students with lower perceived ability to tolerate distress not only reported more frequent cannabis use to cope with negative feelings, but also experienced more severe cannabis-related consequences and cravings. In other words, an inability to sit with anxiety or sadness may push students toward using cannabis as an emotion-regulation crutch, which can inadvertently lead to heavier use and the accumulation of new problems (e.g. academic decline, interpersonal conflicts, health issues)—precisely the kind of cycle described by self-medication theory. Over time, reliance on cannabis might further erode one's natural coping skills, creating a self-perpetuating negative reinforcement loop (30). The present study is particularly interested in examining this dynamic: how do emotion regulation difficulties and low distress tolerance manifest in a university student sample, and are they associated with greater cannabis use and worse mental health? By incorporating *Emotion Regulation Theory, Self-Medication Theory*, and the concept of *distress tolerance* into our framework, we set the stage for understanding cannabis use not simply as a recreational phenomenon but as a potentially compensatory behavior tied to students' psychological experiences.

### Cannabis use and mental health in the university setting

Substantial research in North America and Europe has documented associations between cannabis use and mental health indicators among college students (31). High rates of cannabis consumption have been linked to elevated levels of stress, anxiety, and depression in student samples. One U.S. college survey found that students endorsing cannabis for emotional relief had significantly higher anxiety disorders (including generalized anxiety and panic) and often co-occurring obsessive-compulsive symptoms (32). Similarly, frequent cannabis users report more depressive symptoms on average than non-users, and some evidence points to cannabis use exacerbating suicidal ideation and motivational deficits in youth (33). Conversely, students burdened by depression or anxiety may be especially drawn to cannabis as an escape (34)—reinforcing the vicious cycle noted above. In the academic domain, heavy cannabis use has been associated with lower academic performance and engagement (35). For example, longitudinal studies suggest that adolescents and young adults who use cannabis regularly are more likely to experience lower grades and even higher dropout rates, though these outcomes are often intertwined with other factors like absenteeism or co-occurring substance use (36). It is worth noting that university students today face unprecedented and evolving mental health challenges. Pre-pandemic evidence suggested that approximately 40%−50% of students report moderate to severe symptoms of anxiety or depression (37). More recent research indicates that these rates have increased or remained elevated in the context of the COVID-19 pandemic and its aftermath, with additional contributions from ongoing geopolitical conflicts and global economic instability (e.g., 31). These cumulative stressors have been associated with heightened psychological distress, uncertainty, and vulnerability among young adults, underscoring the importance of examining coping behaviors such as substance use within this broader contemporary context. These internalizing symptoms can themselves impair concentration and academic success, and when students turn to cannabis as a coping mechanism, it may compound the problem. The need to address student mental health and substance use in tandem is therefore critical.

Despite the wealth of research in Western contexts, there is a notable gap in the literature concerning non-Western university students and cannabis use. In Türkiye and similar contexts, empirical data on student substance use and its psychological correlates have been scarce and sometimes inconsistent. Only a handful of studies have quantified the prevalence of cannabis use among Turkish college students, often yielding lower rates than in Western countries [as noted, typically under ~10% (38, 39)]. Moreover, qualitative insights into *why* students in Türkiye might use cannabis—for example, whether for social recreation, experimentation, or self-medication—are virtually absent (40). Cultural factors could significantly shape these motivations and experiences. Given the strong stigma around illicit drugs in Turkish society, students who do use cannabis may experience greater secrecy, shame, or conflict, which could influence their psychological wellbeing differently than students in environments where cannabis use is more normalized (41). Thus, our study addresses a critical need by examining cannabis use and mental health in a non-Western university context, contributing to a more global and culturally nuanced understanding of this issue. We apply a *mixed-methods approach* to capture both broad patterns and deep, personal narratives, recognizing that numbers alone cannot fully illuminate a phenomenon so embedded in personal coping and cultural meaning.

### Rationale for a mixed-methods design

A mixed-methods design was used to provide both breadth and depth in understanding cannabis use among university students. The quantitative component examined associations between cannabis-related problems and psychological variables, whereas the qualitative component explored how students understood and experienced cannabis use in their everyday lives. This design was particularly appropriate because statistical associations alone cannot fully explain the emotional, contextual, and culturally shaped meanings of cannabis use. By integrating both components, the study aimed to generate a more comprehensive understanding of the relationship between cannabis use, psychological distress, and coping in a non-Western university context.

### Study objectives and hypotheses

The present study aimed to examine the associations between cannabis-related problems and key psychological variables—specifically anxiety, depressive symptoms, and distress tolerance—among university students in Türkiye, using a mixed-methods design. In addition to quantifying these relationships, the study sought to explore students' subjective experiences of cannabis use, including its perceived emotional functions, coping-related motivations, and broader academic and interpersonal impacts. By integrating quantitative and qualitative findings, the study aimed to provide a more comprehensive and contextually grounded understanding of how cannabis use and psychological distress interact in this population. Specific objectives included: (1) quantitatively assessing the relationships between cannabis use/problem severity and mental health indicators (symptoms of anxiety and depression, as well as levels of distress tolerance), and (2) qualitatively exploring students' personal narratives around using cannabis—including their motivations (especially any self-medication or emotion-regulation motives), perceived psychological effects, coping behaviors, and the impact of cannabis on their academic and social functioning. By integrating these components, we sought to develop a cohesive understanding and even propose an initial conceptual model of how cannabis use and psychological distress may form a reinforcing cycle in this group.

Based on the theoretical and empirical background discussed, we formulated the following hypotheses for the quantitative phase:

#### Psychological distress hypothesis

Students reporting greater cannabis involvement—particularly those with higher cannabis-related problem severity—will also report higher levels of psychological distress, operationalized as elevated anxiety and depressive symptoms. This reflects our expectation (grounded in self-medication and emotion regulation theories) that problematic cannabis use co-occurs with significant internal distress. We anticipated positive correlations between a cannabis problem index and anxiety/depression scores.

#### Distress tolerance hypothesis

Students with more severe cannabis use/problems will exhibit lower distress tolerance. We hypothesized an inverse relationship between cannabis problem severity and distress tolerance levels. This is in line with prior evidence that individuals who struggle to tolerate emotional discomfort are more prone to heavy or problematic substance use as a coping method.

The qualitative component was designed as an exploratory and interpretive inquiry aimed at identifying recurring themes in students' experiences of cannabis use. Based on the theoretical framework and existing literature, particular attention was given to themes related to emotion regulation, coping processes, and the perceived psychological effects of cannabis use. In particular, we anticipated that students would frequently describe using cannabis as a strategy to manage negative moods, stress, or anxiety (consistent with self-medication theory), and that many would recognize a tension between short-term relief and longer-term consequences (e.g. feelings of dependence, guilt, or academic difficulties). We also expected to uncover contextual factors unique to the Turkish university environment, such as the influence of stigma or peer networks on their experiences. Ultimately, by integrating the quantitative and qualitative findings, our goal was to illuminate the mechanisms by which cannabis use and psychological distress intersect among Turkish students, thereby contributing both theoretically and practically—offering insights that can inform mental health and campus interventions in Türkiye and beyond.

## Methods

### Study design

This study employed a sequential explanatory mixed-methods design (QUAN → qual) to examine the relationship between cannabis use and psychological experiences among university students in Türkiye. In this design, a quantitative phase was conducted first to identify patterns and associations among key variables, followed by a qualitative phase aimed at providing deeper insight into participants' lived experiences and contextualizing the quantitative findings.

Integration of the two components occurred at the interpretation stage, where qualitative findings were used to elaborate and explain quantitative results, allowing for a more comprehensive understanding of the phenomenon under investigation.

### Participants

The quantitative phase included 512 university students recruited from public and private universities in Istanbul, Ankara, and Ìzmir, Türkiye. Standard inclusion criteria were applied, including being 18 years of age or older, current enrollment as an undergraduate or graduate student, and ability to complete self-report questionnaires in Turkish. Exclusion criteria was formally diagnosed psychiatric disorder or current psychiatric treatment. Subclinical symptoms of anxiety and depression, which are common in university populations, were not exclusionary and were assessed dimensionally using self-report measures. Recruitment was conducted using a combination of convenience and snowball sampling through university networks and online platforms. All participants completed a sociodemographic questionnaire and standardized self-report measures assessing cannabis-related problems (CUPIT), depressive symptoms (BDI-II), anxiety symptoms (BAI), and distress tolerance (Distress Tolerance Scale).

The qualitative phase comprised 43 participants purposively selected from among survey respondents who consented to follow-up contact and who demonstrated elevated levels of cannabis-related problems and/or psychological distress in the quantitative phase. Semi-structured interviews were conducted until thematic saturation was achieved. All data were collected anonymously, and no identifying information was obtained.

### Measures

#### Sociodemographic and substance use questionnaire

Participants completed a brief self-administered sociodemographic questionnaire developed for the present study to collect information on age, gender, city of residence (Ìstanbul, Ankara, Ìzmir), academic level, field of study and any diagnosed psychiatric history. The questionnaire also included items assessing lifetime and recent cannabis use (age at first use, frequency of use, and co-use with other substances). These items were used to descriptively characterize the sample and to support interpretation of quantitative and qualitative findings.

#### Cannabis use problems identification test (CUPIT)

Cannabis-related problems were assessed using CUPIT, originally developed by Bashford et al. (42) as a brief screening instrument to identify risky and problematic cannabis use. The CUPIT consists of 16 items assessing frequency of use, impaired control, cannabis-related problems, and perceived risk of harm. The Turkish validation was conducted by Evren et al. (43) in a clinical sample of individuals with cannabis or synthetic cannabinoid use disorder. The Turkish version demonstrated strong internal consistency, with a Cronbach's alpha of.89 for the total scale, and good construct validity with a two-factor structure reflecting impaired control and problems. Higher scores indicate greater cannabis-related problem severity.

#### Beck Depression Inventory–II (BDI-II)

Depressive symptoms were measured using BDI-II, developed by Beck et al. (44) to assess the severity of depressive symptomatology over the past 2 weeks. The scale consists of 21 items rated on a 4-point Likert scale, with higher scores indicating greater depressive severity. The Turkish adaptation and validation were carried out by Kapçi et al. (45), demonstrating high reliability and validity in both clinical and non-clinical samples. Reported Cronbach's alpha values for the Turkish BDI-II typically range from.80 to.90, indicating excellent internal consistency. The BDI-II is widely used in Turkish university student samples.

#### Beck Anxiety Inventory (BAI)

Anxiety symptoms were assessed using BAI, originally developed by Beck et al. (46) to measure the severity of common anxiety symptoms. The BAI comprises 21 items, each rated on a 4-point Likert scale, focusing primarily on somatic and subjective anxiety symptoms experienced during the past week. The Turkish validation was conducted by Ulusoy et al. (47), who reported strong psychometric properties in psychiatric and community samples. The Turkish version has demonstrated high internal consistency, with Cronbach's alpha coefficients typically around.93, and good convergent validity with other anxiety measures.

#### Distress Tolerance Scale (DTS)

Distress tolerance was assessed using DTS, developed by Simons and Gaher (48) to measure individuals' perceived capacity to withstand negative emotional states. The DTS consists of 15 items rated on a 5-point Likert scale and yields a total score as well as four subscales: tolerance, absorption, appraisal, and regulation. The Turkish adaptation and validation were conducted by Akin et al. (49) in a large sample of university students. The Turkish version demonstrated good construct validity and acceptable reliability, with a Cronbach's alpha of.82 for the total scale and subscale alphas ranging from.61 to.71. Higher scores reflect greater perceived ability to tolerate psychological distress.

#### Qualitative interview protocol

A semi-structured qualitative interview protocol was developed to explore in depth the psychological experiences associated with cannabis use among university students who demonstrated elevated levels of cannabis-related problems and/or psychological distress in the quantitative phase. The protocol was informed by the study's quantitative findings (CUPIT, BDI-II, BAI, DTS) and by existing literature on substance use, emotional regulation, and coping in young adults. The interview guide was designed to be flexible, allowing participants to elaborate freely while ensuring coverage of key domains relevant to the research aims.

Interviews focused on participants' cannabis use trajectories, subjective psychological effects, emotional and cognitive experiences, and coping-related functions of use, particularly in relation to distress tolerance, anxiety, and mood states. Core domains included: (1) initiation and patterns of cannabis use; (2) perceived short- and long-term psychological effects of cannabis; (3) motivations for use, including use in response to stress, anxiety, or negative mood; (4) experiences of loss of control, ambivalence, or perceived dependence; (5) perceived impact of cannabis use on academic functioning, interpersonal relationships, and daily life; and (6) meanings attributed to cannabis use within participants' broader life context.

Sample guiding questions included: “Can you tell me about when and how you first started using cannabis?”; “How does cannabis affect you emotionally or psychologically when you use it?”; “In what situations do you feel more inclined to use cannabis?”; “How do you usually cope with distress or anxiety, and what role does cannabis play in this process?”; “Have you noticed any changes in yourself—emotionally, cognitively, or socially—over time in relation to your cannabis use?”; and “How do you see your cannabis use fitting into your life at this stage?”

Probing questions were used to clarify meanings, explore examples, and deepen reflection (e.g., “Can you give an example?”, “How did that make you feel?”, “What happened next?”). Interviews were conducted in Turkish, audio-recorded with participants' consent, and transcribed verbatim. Data collection continued until thematic saturation was reached, defined as the point at which additional interviews no longer yielded novel themes relevant to the study aims.

### Data analysis

#### Quantitative analysis

Quantitative data were analyzed using IBM SPSS Statistics (Version 28). Descriptive statistics (means, standard deviations, frequencies, and percentages) were computed to summarize sociodemographic characteristics, cannabis-related problem severity (CUPIT), depressive symptoms (BDI-II), anxiety symptoms (BAI), and distress tolerance (Distress Tolerance Scale). Internal consistency reliability of the scales was evaluated using Cronbach's alpha coefficients. Bivariate associations between cannabis-related problems and psychological variables were examined using Pearson correlation analyses. These analyses were conducted to characterize the psychological profile of the sample and to identify patterns that informed selection of participants for the qualitative phase. Statistical significance was set at *p* < 0.05.

#### Qualitative analysis

Qualitative interview data were analyzed using reflexive thematic analysis (Maxqda), following the six-phase framework proposed by Braun and Clarke (50). Interview transcripts were read repeatedly to achieve data familiarization, after which initial codes were generated inductively to capture meaningful units related to psychological experiences of cannabis use. Codes were then organized into candidate themes, which were reviewed, refined, and defined through iterative comparison across transcripts. Analysis emphasized participants' subjective meanings, emotional and cognitive experiences, and perceived functions of cannabis use in relation to distress, anxiety, and mood regulation. To enhance analytic rigor, coding and theme development were conducted iteratively, with constant reference to the original data and ongoing reflexive consideration of researchers' assumptions. Data collection and analysis proceeded concurrently, and thematic saturation was considered achieved when no new conceptually relevant themes emerged.

#### Mixed-methods integration

Integration of quantitative and qualitative components occurred at the design, sampling, and interpretation levels. First, results from the quantitative phase guided criterion-based purposive sampling for the qualitative phase, with interviews conducted among participants exhibiting elevated CUPIT scores and higher psychological symptom burden. Second, during interpretation, qualitative themes were used to explain and contextualize quantitative patterns, particularly associations between cannabis-related problems, emotional distress, and distress tolerance. Findings from both components were compared and synthesized to identify areas of convergence, complementarity, and expansion, thereby providing a more comprehensive understanding of cannabis use and psychological experiences among university students. Quantitative results provided breadth and pattern identification, while qualitative findings offered depth and explanatory insight into the lived experiences underlying those patterns.

Gender was recorded as a demographic variable; however, no subgroup analyses were conducted based on gender.

## Results

### Sample characteristics

The quantitative sample (*N* = 512), slightly over half of participants were female (55.9%), and the mean age was 21.8 years (SD = 2.4). Most were undergraduate students (88% undergraduate vs. 12% graduate), and a range of academic fields were represented (approximately 39% in social sciences, 24% in health sciences, 21% in engineering/natural sciences, and 16% in other fields). The majority were not formally employed during their studies (about 73% unemployed), consistent with a student sample. By design, all participants had used cannabis at least once in their lifetime; notably, 80% reported using cannabis at least once per month in the past 3 months, including about half who used weekly or more frequently (33.6% weekly and 17.6% daily/almost daily). This indicates that while all participants had some history of use, a substantial subset were regular users, providing variability in use patterns.

From this survey sample, a purposive subsample of 43 students was selected for the qualitative phase. These individuals were chosen based on exhibiting elevated cannabis-related problem severity (high CUPIT scores) and, in many cases, greater psychological distress (e.g. higher depression/anxiety scores) and lower distress tolerance in the survey. The qualitative subsample had a mean age of 22.4 years (SD = 2.6) and included 19 females and 24 males, roughly mirroring the gender balance of the quantitative sample. Participants in the subsample were drawn from the same university sites (about 42% from Istanbul, 33% Ankara, 25% Ìzmir), and all had high cannabis problem scores by definition. Consistent with the selection criteria, the majority of these interviewees (72%) were frequent users (weekly or more often), suggesting that the qualitative data pre-dominantly reflect experiences of heavier cannabis involvement.

Of the initial respondents, **4 cases** were excluded due to incomplete or invalid questionnaire responses. The final analytic sample consisted of *N* = 512, with minimal missing data that did not require imputation.

### Quantitative results

#### Descriptive statistics of study variables

The participants reported, on average, moderate levels of cannabis-related problems alongside notable symptoms of anxiety and depression, and moderate perceived distress tolerance (see Table 1). For instance, the mean score on CUPIT was 24.6, suggesting a mid-range level of cannabis problem severity in this student sample. Mean scores on the mental health measures indicated mild to moderate psychological symptomatology on average: BDI-II mean was 17.9 (SD = 9.6) and BAI mean was 16.4 (SD = 10.2), both of which are elevated relative to community norms but not extreme. The average score on DTS was 41.2 (SD = 8.7), indicating a moderate perceived ability to tolerate emotional distress. Notably, score distributions for all measures spanned a wide range without severe floor or ceiling effects, reflecting ample variability.

**Table 1:** Descriptive Statistics of Main Study Variables (*N* = 512).

Measure	Mean	SD	Possible range
CUPIT (total)	24.6	7.8	0–64
BDI-II (total)	17.9	9.6	0–63
BAI (total)	16.4	10.2	0–63
Distress Tolerance Scale (total)	41.2	8.7	15–75
Tolerance	10.1	2.6	5–25
Absorption	9.6	2.4	4–20
Appraisal	11.3	3.1	5–25
Regulation	10.2	2.8	5–25

Higher scores indicate greater cannabis-related problem severity (CUPIT), higher depressive symptoms (BDI-II), higher anxiety symptoms (BAI), and greater perceived distress tolerance (Distress Tolerance Scale).

#### Associations between cannabis-related problems and psychological variables

Bivariate analyses revealed a clear pattern of significant associations between cannabis-related problem severity and psychological distress variables (see Tables 2a, b). Higher CUPIT scores were moderately positively correlated with both depressive symptoms (BDI-II) and anxiety symptoms (BAI; *r* ≈0.45–0.48, *p* < 0.001). In other words, students who reported more severe cannabis-related problems tended to also report greater emotional distress in terms of depression and anxiety. Conversely, CUPIT scores were negatively correlated with distress tolerance (*r* ≈ −0.39, *p* < 0.001), indicating that those with more cannabis problems generally reported a lower ability to withstand negative emotional states. These findings suggest an inverse relationship between heavy/problematic cannabis use and one's capacity to tolerate psychological distress.

**Table 2a:** Pearson correlations among cannabis-related problems, psychological symptoms, and distress tolerance (*N* = 512).

Variable	1	2	3	4
1. CUPIT (total)	—			
2. BDI-II (total)	0.48^**^	—		
3. BAI (total)	0.44^**^	0.62^**^	—	
4. Distress tolerance (total)	−0.39^**^	−0.51^**^	−0.47^**^	—

CUPIT, cannabis use problems identification test; BDI-II, Beck Depression Inventory–II; BAI, Beck Anxiety Inventory.

Higher distress tolerance scores indicate greater perceived ability to tolerate psychological distress.

^**^p < 0.001.

**Table 2b:** Pearson correlations between cannabis-related problems and distress tolerance subscales (*N* = 512).

Variable	1	2	3	4	5
1. CUPIT (total)	—				
2. Tolerance	−0.34^**^	—			
3. Absorption	−0.37^**^	0.56^**^	—		
4. Appraisal	−0.29^**^	0.48^**^	0.52^**^	—	
5. Regulation	−0.32^**^	0.51^**^	0.49^**^	0.54^**^	—

CUPIT, cannabis use problems identification test.

Distress tolerance subscales are derived from the Distress Tolerance Scale (Simons and Gaher).

Higher scores reflect greater distress tolerance across domains.

^**^p < 0.001.

The pattern extended to inter-relationships among the psychological measures themselves: depressive and anxiety symptom scores were strongly positively correlated with each other (*r* ≈0.62, *p* < 0.001), as expected given the comorbidity of anxiety and depression in young adults. Both depression and anxiety were also significantly inversely correlated with distress tolerance (*r* = −0.51 and *r* = −0.47, respectively, *p* < 0.001), consistent with the idea that higher emotional symptom burden co-occurs with a lower perceived ability to cope with distress. Furthermore, an expanded correlation analysis examining the four DTS subscales provided a more nuanced view of distress tolerance in relation to cannabis use. Higher cannabis problem scores were associated with difficulties across all facets of distress tolerance: specifically, greater problems correlated with *lower tolerance* for emotional upset (*r* = −0.34, *p* < 0.001), a *stronger tendency to absorb* or become overwhelmed by negative emotions (i.e. higher absorption; *r* = −0.37, *p* < 0.001), more *negative appraisals* of emotional distress (*r* = −0.29, *p* < 0.001), and poorer *emotional regulation* in distressing situations (*r* = −0.32, *p* < 0.001). All of these correlations were statistically significant, underscoring a robust relationship between problematic cannabis involvement and reduced distress tolerance across multiple dimensions. Taken together, the quantitative results outline a coherent profile: students with more severe cannabis use problems tend to experience more intense anxiety/depression symptoms and have diminished resilience in the face of stress. These broad associations set the stage for the qualitative findings, which delve into the lived experiences behind these numbers.

### Qualitative results

#### Overview of thematic structure

The in-depth interviews with 43 participants provided rich, contextualized insight into how and why students use cannabis and how it connects to their psychological experiences. Through reflexive thematic analysis, we inductively identified four overarching themes that recur across participants' accounts: (1) Psychological Effects of Cannabis Use; (2) Cannabis Use as a Distress Regulation Strategy; (3) Perceived Loss of Control and Ambivalence; and (4) Academic and Interpersonal Consequences. Each theme comprised several subthemes that capture variations in individual experiences (for example, different emotional responses or situational triggers within a broader theme). No new themes emerged as coding progressed, indicating that thematic saturation was achieved with the sample.

#### Theme 1: psychological effects of cannabis use

Participants consistently described a range of immediate psychological and emotional effects resulting from cannabis use. A dominant pattern was the experience of short-term emotional relief and relaxation. Many students reported that shortly after using cannabis, they felt an initial wave of calmness or emotional numbing that helped ease feelings of stress or anxiety. It was common for them to describe a sense of “slowing down” in the mind, where racing or ruminative thoughts were temporarily quieted. This mental calming often translated into physical and emotional relaxation—for example, feeling less tense or experiencing a lightened mood pressure for a brief period. Alongside this emotional relief, participants noted distinct cognitive and perceptual changes. These included *slowed or altered thinking*, trouble concentrating on complex tasks, and a sensation of mental detachment or “drifting.” Some described feeling mentally distant or having a shifted perspective on their surroundings. Notably, these cognitive effects were double-edged: in certain contexts they were perceived as soothing (providing a break from overthinking or intense focus), whereas in other contexts they were experienced as impairing (for instance, making studying or driving difficult due to reduced concentration).

Another notable aspect of cannabis's psychological impact was its tendency to modulate the intensity of emotions, albeit in varying ways depending on dose and context. Several participants observed that cannabis could amplify certain affective states or, conversely, blunt them. On the positive side, moderate use often enhanced feelings of comfort or contentment—students mentioned feeling more at ease or even pleasantly cheerful in relaxed settings. However, negative or undesired emotional reactions were also reported, particularly with heavier use or higher-potency cannabis. For example, some participants recounted episodes of increased irritability or a numb, flattened emotional state after frequent use, describing feeling “disconnected” or lacking emotional responsiveness. A subset of individuals—especially those who used large quantities or had very frequent sessions—experienced transient bursts of paranoia or anxiety during or after using, such as sudden feelings of being unsafe or overly self-conscious. Participants generally recognized these adverse reactions as dose-dependent and highly individual. Indeed, some noted that the effects of cannabis evolved over time: what began as a reliably relaxing experience could become less predictable or less positive as their use became more regular. For instance, one student explained that early in their use, cannabis always made them calm, but after months of near-daily use, it sometimes left them feeling “numb and anxious at the same time.” Such accounts highlight that the psychological effects of cannabis are not uniform—while short-term relief is common, the overall emotional impact can shift with prolonged or heavy use, introducing new challenges like emotional blunting or exacerbated anxiety for some individuals (see Table 3 for quotations exemplifying these effects).

**Table 3:** Theme 1: psychological effects of cannabis use—subthemes and exemplar quotations (*n* = 43).

Subtheme	Description	Exemplar quotation
Emotional relief and relaxation	Cannabis use described as producing calmness and temporary emotional relief	“When I smoke, my mind slows down. I feel calmer, like the pressure eases for a while.”
Cognitive and perceptual changes	Alterations in thinking, attention, and perception following use	“I notice that my thoughts become slower and less focused. Sometimes I just drift and lose track of what I was doing.”
Emotional amplification or blunting	Intensification or dulling of emotions depending on context and dose	“Sometimes it makes me feel more comfortable and relaxed, but other times I feel kind of numb, like I'm disconnected.”
Anxiety-related effects	Cannabis-induced reduction or increase in anxiety symptoms	“At first it helps with my anxiety, but if I use too much, I can feel paranoid or uneasy.”

#### Theme 2: cannabis use as a distress regulation strategy

Across the interviews, students frequently discussed using cannabis in a purposeful way to manage psychological distress. Cannabis was widely portrayed as a deliberate coping tool—a means to alleviate stress, anxiety, or low mood in the short term. Many participants said they tended to reach for cannabis during times of high tension, emotional overwhelm, or after a particularly stressful day, as a way to “switch off” their racing thoughts and worries. They described the drug's effects as helping to “turn down the volume” of negative emotions or create a temporary emotional buffer. For instance, a student who often felt anxious about academic pressures noted that smoking cannabis in the evening allowed them to detach from those concerns, experiencing a sense of momentary peace or indifference about problems that normally felt urgent. In this way, immediate relief from anxiety or sadness was a powerful motivator for use: participants found that cannabis could quickly soothe feelings of nervousness, lift a heavy mood, or induce a sense of emotional stability when they felt on the verge of losing control.

However, the interviews also revealed that this reliance on cannabis for relief often involved an avoidance-based coping pattern. Students candidly described using cannabis as a way to escape or postpone dealing with negative internal states. Rather than confronting the source of their stress or sadness, they would use cannabis to numb themselves or push away uncomfortable feelings. Several participants acknowledged that getting high served as a form of emotional avoidance—a conscious decision to not “sit with” painful emotions like anxiety, grief, or frustration. For example, one participant shared that whenever they felt a wave of panic or depressive thoughts coming on, their instinct was to smoke cannabis immediately so they “wouldn't have to face those feelings for a while.” This approach provided short-term relief but did nothing to resolve the underlying issues, a fact that some participants recognized with hindsight. Indeed, a recurrent sentiment was that cannabis offered a *temporary* reprieve; once the intoxicating effects wore off, the original problems or emotions often remained unchanged (and in some cases, were waiting with even greater intensity).

Notably, participants' narratives often tied into the concept of distress tolerance, aligning with the quantitative findings. Students with higher self-reported anxiety described cannabis as a crucial tool to “quiet” their excessive worry or physical agitation, whereas those prone to low mood emphasized how it could momentarily lift the weight of sadness or emptiness. At the same time, a number of participants reflected on how their over-reliance on cannabis might have eroded their natural capacity to cope with distress when not using. They admitted that the more they turned to cannabis as a quick fix for stress or bad moods, the more they felt incapable of handling even minor stressors without it. One participant noted that after months of frequent use, they found themselves “less and less able to calm down” or regain emotional equilibrium on their own, compared to before they started using regularly. Such observations suggest that, in these students' experiences, habitual cannabis use may undermine the development or utilization of healthy coping skills, creating a vicious cycle wherein cannabis becomes increasingly necessary to manage emotions. In summary, cannabis was commonly viewed and utilized as an expedient (if short-term) strategy for emotion regulation, allowing students to avoid negative feelings in the moment, even as this strategy potentially reinforced a lower tolerance for distress over time. Representative quotations illustrating these subthemes are presented in Table 4.

**Table 4:** Theme 2: cannabis use as a distress regulation strategy—subthemes and exemplar quotations (*n* = 43).

Subtheme	Description	Exemplar quotation
Use for emotional relief	Cannabis used to reduce anxiety, stress, or negative mood	“When I feel overwhelmed, I smoke to calm down. It helps me forget things for a while.”
Avoidance coping	Use to escape or suppress distressing thoughts and emotions	“I don't want to deal with what I'm feeling, so I just smoke and push it away.”
Situational triggers	Increased use during stress, academic pressure, or emotional strain	“After a stressful day or exams, I feel like I need it just to relax.”
Perceived short-term benefit	Temporary emotional relief without long-term resolution	“It helps in the moment, but the problems are still there later.”

#### Theme 3: perceived loss of control and ambivalence

Many participants' accounts conveyed a growing difficulty in controlling their cannabis use, accompanied by mixed feelings about their continued use. Students often described struggling to regulate the frequency or amount of cannabis consumed, even when they set intentions to cut back. It was not uncommon for individuals to share anecdotes of trying to limit their use to certain days or contexts (for example, “only on weekends” or “only after exams”), only to find those boundaries gradually eroding. A number of participants noticed an escalation of use over time—what might have started as an occasional recreational activity had, for some, turned into a near-daily habit. This loss of control was sometimes characterized by a sense of use becoming almost automatic or compulsive. One student explained that they “realized I was reaching for a joint without even deciding to”, highlighting how ingrained the behavior had become in their routine. Importantly, although few participants explicitly labeled themselves as “addicted,” they often acknowledged that their level of use had become problematic or hard to manage in their own view. They expressed concern that cannabis was beginning to dominate their time, thoughts, or money, indicating that their relationship with the drug was no longer entirely under their voluntary control.

Alongside these struggles with control, participants universally expressed ambivalent attitudes toward cannabis. They simultaneously valued and feared their cannabis use, and this internal conflict was a prominent theme. On one hand, students readily pointed out the perceived benefits they got from cannabis—such as emotional relief, relaxation, enhanced social experiences, or creativity—which made them reluctant to give it up entirely. On the other hand, they were also acutely aware of the negative consequences and personal drawbacks that came with heavy use. This led to complex feelings of guilt, worry, and rationalization. For example, a participant might mention how cannabis helped them calm their nerves, but in the next breath acknowledge feeling guilty about relying on a substance and anxious about potential health or academic repercussions. Many described an internal dialogue of justification and regret: *justifying* their use because “it helps me cope” or “everyone I know does it,” yet feeling *regretful* or worried when reflecting on how it was affecting their life or could affect their future. This tug-of-war between the enjoyment and relief cannabis provided and the concerns about its impact created a persistent psychological tension. Several participants used phrases like “love-hate relationship” or “my friend and enemy” to describe cannabis, underscoring the depth of their ambivalence. In essence, cannabis use was experienced as both helpful and harmful simultaneously, leaving students uncertain about how to view it or what role it should play in their lives. This ambivalence often accompanied, and perhaps exacerbated, the difficulty in cutting down—as long as the benefits remained attractive, quitting or reducing use became an emotionally fraught decision. Illustrative quotations corresponding to these experiences of loss of control and ambivalence are presented in Table 5.

**Table 5:** Theme 3: perceived loss of control and ambivalence—subthemes and exemplar quotations (*n* = 43).

Subtheme	Description	Exemplar quotation
Loss of control	Difficulty regulating frequency or amount of use	“I tell myself I won't smoke, but I end up doing it anyway.”
Escalation of use	Gradual increase in frequency or dependency	“It used to be occasional, now it's almost every day without realizing it.”
Ambivalence toward use	Simultaneous recognition of benefits and harms	“It helps me relax, but I also know it's causing problems.”
Guilt and concern	Feelings of regret, worry, or internal conflict	“Sometimes I feel guilty, like I'm depending on it too much.”

#### Theme 4: academic and interpersonal consequences

Participants' narratives also illuminated various functional consequences of cannabis use, particularly in their academic performance and social relationships. In the academic domain, many students reported that frequent cannabis use had detrimental effects on their studies over time. Common difficulties included impaired concentration, decreased motivation, and poor time management when under the influence or in the aftermath of use. For instance, some mentioned that after an evening of getting high, they found it much harder to wake up for morning classes or to focus on assignments the next day. Others noted a pattern of procrastination linked to cannabis—using it to relax would sometimes spiral into lost study time or neglected coursework. These academic impacts were often described as cumulative rather than immediate. Rather than a single instance of intoxication dramatically lowering a test score, it was the pattern of regular use that slowly eroded their class attendance, participation, and overall performance. One participant reflected that during a semester of heavy use, they “didn't realize how much it was affecting me until I saw my grades drop at the end,” suggesting that the academic fallout became apparent only in retrospect. Not all students experienced severe academic consequences, but even those who maintained acceptable grades voiced concerns that cannabis made it harder to perform at their *personal best*. In summary, academic functioning tended to suffer in subtle ways with persistent cannabis use, through reduced productivity and engagement, which over time could snowball into significant performance issues.

In terms of interpersonal life, the effects of cannabis use were mixed but often leaned toward the negative as use became more intensive. A few participants felt that cannabis had a social facilitation effect in certain contexts—for example, using together with friends sometimes enhanced bonding or made social gatherings feel more relaxed and enjoyable. However, more commonly, students reported that heavy cannabis use led to some degree of social withdrawal or strain on relationships. Some described becoming more isolated over time, preferring to spend time alone or only with friends who also used cannabis, which narrowed their social circle. This could create distance between them and friends who did not use or who disapproved of use. Several participants mentioned experiencing tension or conflict with family members or non-using friends due to their cannabis habits. For instance, hiding their use or lying about it to parents and close friends caused feelings of guilt and deteriorated trust once the use was discovered. Other students noted they had less interest in social activities that did not involve cannabis, leading to missed opportunities to engage in campus life or maintain relationships outside of the “stoner” culture. Thus, while cannabis occasionally served as a social lubricant within a like-minded peer group, its overall impact as described by participants was more often to distance them from important aspects of social and academic life. Over time, these interpersonal shifts—whether it was drifting away from certain friendships, increased conflict at home, or a general retreat from social involvement—became part of the cost of their cannabis use. Such consequences were often intertwined with the psychological aspects noted above (for example, feeling anxious or guilty about letting others down academically or socially, yet continuing to use, thereby feeding into further distress).

### Mixed-methods integration

#### Convergence of quantitative and qualitative findings

When examining the survey results and interview findings together, there was a strong convergence between the quantitative and qualitative evidence. The patterns observed in the statistical data were vividly reflected in the personal narratives of participants. Quantitatively, These findings may reflect greater cannabis-related problem severity was associated with higher levels of anxiety and depression, and with lower distress tolerance. The qualitative accounts confirm and lend insight into these associations: students frequently discussed their cannabis use *in the context of coping with anxiety, low mood, and stress*, and many described feeling less capable of handling distress without cannabis. For example, the survey correlation showing that higher CUPIT scores correlated with higher BDI-II/BAI scores aligns with numerous interviewees' statements that they used cannabis as an outlet or self-medication for persistent worry, sadness, or emotional pressure. Likewise, the inverse relationship between cannabis problems and distress tolerance (quantitatively indicated by a negative correlation) was mirrored in participants' stories of relying on cannabis because they struggled to tolerate negative feelings on their own. In essence, both data strands independently point to the same overarching picture: students who are more deeply involved in problematic cannabis use are also those experiencing significant emotional distress and difficulty coping with that distress without substance use. There were no glaring contradictions between what the numbers showed and what the students described. On the contrary, the qualitative narratives reinforced the credibility of the quantitative findings by providing real-life examples of the very dynamics suggested by the survey data. This convergence strengthens our confidence in the results—the fact that the statistical associations have clear echoes in lived experience suggests that the relationships observed are not spurious. Rather, they likely reflect genuine underlying connections between cannabis use and psychological distress in this population (see Table 6 for a joint display linking quantitative results with qualitative themes).

**Table 6:** Joint display linking quantitative findings and qualitative themes.

Quantitative finding	Corresponding qualitative theme	Illustrative qualitative meaning
Higher CUPIT scores associated with higher BDI-II and BAI scores	Cannabis use as distress regulation	Cannabis used to manage anxiety, stress, and low mood
Higher CUPIT scores associated with lower distress tolerance	Emotional avoidance and reliance on cannabis	Reduced perceived ability to tolerate distress without using cannabis
Intercorrelations among distress, anxiety, and cannabis problems	Loss of control and ambivalence	Simultaneous perceived benefits and negative consequences of use
Elevated cannabis-related problems	Academic and interpersonal consequences	Functional impairments emerging over time

Beyond simply confirming the quantitative results, the qualitative findings expanded upon and enriched our understanding of the underlying psychological mechanisms linking cannabis use with mental health. The survey data by itself indicated that cannabis problems and psychological distress go hand-in-hand, but it could not explain *how* or *why* this is the case. The interviews filled in these gaps. Specifically, qualitative accounts revealed a plausible process driving the correlations: students often turned to cannabis as a short-term remedy for feelings of distress, which helps explain why those with higher anxiety or depression were more likely to engage in heavy or problematic use (i.e. their use was motivated by distress relief rather than purely recreational). Participants described this process in their own words, noting that they smoked to calm their nerves or lift their mood when feeling down—thereby elucidating the *meaning* behind the positive correlation of CUPIT with BAI/BDI-II scores. Moreover, the interviews shed light on why higher cannabis problem severity might co-occur with lower distress tolerance: frequent reliance on cannabis to manage emotions can lead to a diminished capacity to tolerate distress unaided. As several students reflected, by constantly escaping into cannabis use instead of facing difficulties, they found themselves less practiced or confident in dealing with adversity without a substance. This insight from the qualitative data helps explain the *mechanism* behind the quantitative inverse relationship—it suggests that problematic cannabis use is both a symptom and a cause of poor distress tolerance, creating a feedback loop that the survey alone could only hint at.

The qualitative findings also introduced additional dimensions that were not captured by our quantitative measures, thereby broadening the scope of what we understand about cannabis use in this group. For instance, participants' expressions of ambivalence and loss of control provide context for the emotional burden associated with cannabis problems. The survey identified that psychological distress and cannabis problems are linked, and the interviews suggest one reason may be that students feel conflicted and guilty about their heavy use, which in itself can fuel stress and depressive feelings. A student who cannot control their use may experience frustration or lowered self-esteem, potentially exacerbating anxiety or depression—a nuance that quantitative measures of symptoms and use severity alone could not reveal. Additionally, the theme of academic and interpersonal consequences extends our understanding of “problems” beyond the psychological symptoms quantified in the survey. The CUPIT measure mostly captures aspects of dependence and personal problems related to use, but students' reports of declining academic performance and strained relationships illustrate real-world functional impairments associated with their cannabis use. These impairments, while not directly measured in the quantitative phase, are consistent with a high level of problem severity and likely contribute to overall distress (for example, academic failure or social conflict can worsen one's mental health, creating further reasons to use cannabis, and so on). The qualitative findings extend the quantitative results by providing contextual insight into how these associations are experienced and interpreted by students.

**Figure 1:**
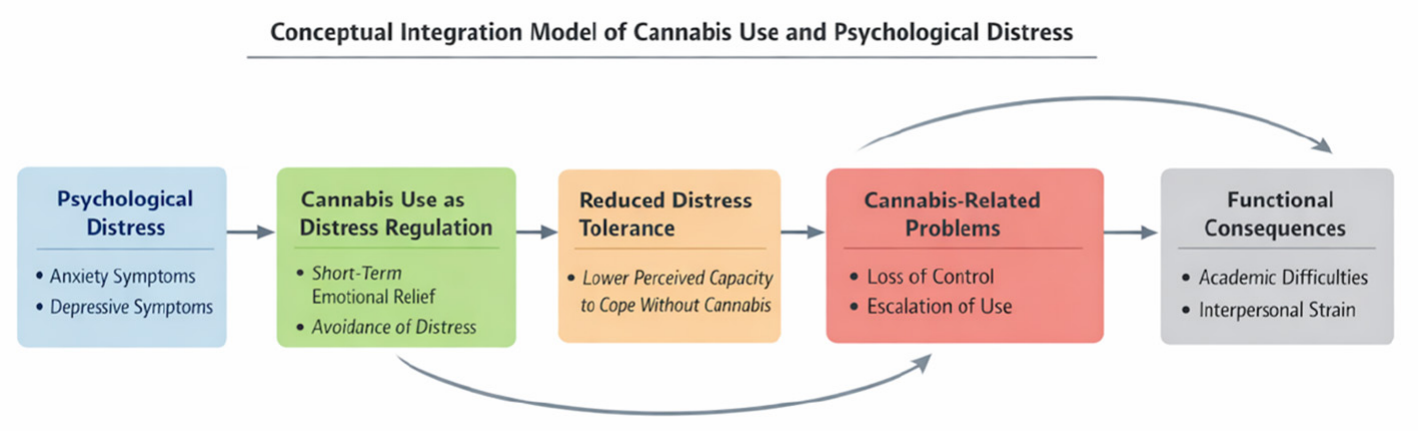
Conceptual integration model linking cannabis-related problems with psychological distress and distress tolerance.

The figure 1 illustrates the convergence of quantitative and qualitative findings. Higher psychological distress (anxiety and depressive symptoms) is associated with cannabis use as a short-term distress regulation strategy. Repeated reliance on cannabis for emotional relief corresponds with lower perceived distress tolerance, which in turn is linked to greater cannabis-related problems and functional consequences in academic and interpersonal domains. The model reflects patterns identified across both quantitative associations and qualitative themes.

## Discussion

Our mixed-methods findings paint a convergent and compelling picture of how cannabis use interweaves with psychological experiences in this sample of Turkish university students. The findings suggest that cannabis use among university students is closely linked to underlying emotional distress and notably lower distress tolerance (51, 52), compared to their peers. These associations, while cross-sectional, align strongly with prior research and theoretical expectations. The qualitative interviews vividly illustrated these patterns. A dominant theme was “cannabis use as distress regulation,” with many participants describing in their own words that they smoke to “calm my nerves,” “forget my problems,” or “lift my mood when I'm down.” Such narratives directly echo the Self-Medication Theory and negative reinforcement framework (53). This convergence between data strands strengthens the credibility of our results. The qualitative accounts helped to explain the quantitative correlations: for example, why would higher cannabis problem scores be linked to higher anxiety/depression? Because, as participants revealed, those with greater emotional distress were often the ones turning to cannabis frequently as an outlet. One interviewee might describe intense worry or sadness leading them to roll a joint nightly; another might recount how feeling “on edge” after a bad exam or during family conflicts triggers their urge to use. These personal examples illustrate the mechanism of negative reinforcement: cannabis provides short-term relief from distress, thereby reinforcing further use in similar emotional contexts (54, 56). This finding is highly consistent with other studies of young adults—research has shown that those who use cannabis to cope indeed report higher rates of anxiety disorders and often an escalation of use frequency and problems over time (55). Our study extends such findings into a non-Western cultural setting, demonstrating that this dynamic appears to transcend cultural differences.

Perhaps even more intriguing was the interplay between low distress tolerance and cannabis use that emerged. These findings indicate that students who scored low on the distress tolerance scale—essentially those who admitted “I can't handle feeling upset or distressed for long”—were disproportionately represented among heavy or problematic cannabis users. Interview data gave life to this pattern: participants with intense cannabis involvement often described themselves as “not good at dealing with stress” or “overwhelmed easily,” and they cited cannabis as a go-to escape mechanism when emotional pressure built up. This aligns with accumulating evidence that distress intolerance is a key vulnerability factor for substance misuse. As Buckner et al. (29) note, individuals with low distress tolerance are especially likely to crave cannabis and use it when experiencing negative affect. Our participants' stories support this: one student described suffering panic-like feelings before presentations and using cannabis beforehand to blunt her anxiety; another mentioned he “couldn't stand” the loneliness and frustration during pandemic lockdowns, so he increased his cannabis intake. These accounts are consistent with a reinforcing cycle in which low distress tolerance increases reliance on cannabis for short-term relief, while repeated use may limit the development of effective coping strategies.

Drawing both strands together, a more integrative understanding emerges of how cannabis use and psychological distress interconnect in a cyclical fashion. The quantitative results provided the outline of this cycle (high distress ↔ high cannabis problems ↔ low distress tolerance), and the qualitative findings fleshed it out with narrative detail. To summarize, and as depicted in Figure 1, we propose the following integrative model: Elevated psychological distress (heightened anxiety and depressive symptoms) can drive students to use cannabis as a maladaptive coping mechanism for immediate relief. This short-term relief, while reinforcing continued use, does not resolve underlying problems and over time may erode the individual's inherent tolerance for distress. Lower distress tolerance then leaves the person increasingly dependent on cannabis to handle even minor stress, which tends to increase the frequency and quantity of use. As cannabis use escalates, cannabis-related problems accumulate—including potential cognitive, academic, and social repercussions—which can further contribute to emotional distress. This creates a self-perpetuating cycle or feedback loop: distress fuels heavier cannabis use, and heavy use in turn breeds lower coping ability and additional life problems, feeding back into greater overall distress. Both our quantitative data (showing the statistical links in this cycle) and our qualitative data (providing experiential evidence of each link) support this dynamic model. In conclusion, the integrated findings paint a coherent picture in which problematic cannabis use among university students is closely intertwined with their psychological wellbeing. Cannabis use frequently functions as a short-term coping strategy, but may contribute to a reinforcing cycle of reduced coping capacity and increased psychological distress. This nuanced understanding, arising from the convergence of quantitative and qualitative evidence, offers a foundation for developing more effective interventions and supports for young people struggling at the intersection of cannabis use and mental health.

Another important qualitative theme was ambivalence and loss of control. Many participants expressed a conflicted attitude toward their cannabis use—they acknowledged both pros and cons. For example, a student might say cannabis helps them sleep or feel less depressed, yet in the same breath admit that “I'm smoking more than I should” or “I worry I'm becoming too dependent on it.” Such ambivalence is typical in substance use narratives and adds a psychological burden of guilt or anxiety. In our study, these feelings of conflict and self-blame appeared to contribute to distress as well. Some students described shame in hiding their use from family (given the stigma in Türkiye) or guilt that “I'm wasting time and hurting my potential.” This emotional burden can further exacerbate mental health issues and may help explain part of the association between cannabis problems and depressive symptoms. In addition to internal distress, participants described functional consequences of heavy use, including academic difficulties and interpersonal strain. For instance, students reported missing classes or performing poorly, which increased stress about their academic future, while others described conflicts with parents or partners that led to feelings of isolation or failure. Although our quantitative survey did not directly measure academic performance or relationships, it did capture general problem severity (via CUPIT), and the interviews suggest that such functional impairments are key components of these problems. This extends the interpretation of our quantitative findings: higher CUPIT scores may reflect not only internal symptoms but also broader impacts on functioning. These patterns are consistent with prior research indicating that cannabis use among university students is associated with poorer academic outcomes and strain in social roles, particularly at higher levels of use.

Our results are theoretically and empirically consistent with a growing body of literature indicating that cannabis use among young adults is closely associated with psychological distress, emotion regulation difficulties, and coping-motivated use [e.g., (9, 10, 20, 55–58)]. From an Emotion Regulation Theory perspective, cannabis use may function as an external strategy for mood modulation, albeit a short-term and ultimately maladaptive one. The Self-Medication Theory is also supported by participants' accounts, as students described using cannabis to manage anxiety, stress, or depressive feelings, consistent with Khantzian's notion that substance use reflects attempts to alleviate psychological distress. The concept of distress tolerance further helps explain variability in use patterns, with individuals who have lower tolerance for emotional discomfort appearing more vulnerable to problematic use. These findings are consistent with prior research; for example, Lucke et al. (9) reported that young adults who use cannabis exhibit higher emotion dysregulation and coping-related motives. This alignment across studies suggests that the psychological drivers of cannabis misuse may be broadly similar across cultural contexts. At the same time, our findings highlight the importance of context, as stigma and secrecy in more conservative settings may intensify the psychological burden associated with use. Rather than contradicting existing literature, our study provides a more contextually grounded understanding of these established associations.

### Clinical and psychosocial implications

The findings of this study highlight the need to address cannabis use and psychological distress as interconnected processes in university populations. For students, the results suggest that using cannabis as a primary coping strategy may provide short-term relief but can contribute to worsening distress and reduced coping capacity over time. This underscores the importance of promoting alternative emotion regulation strategies, such as mindfulness, physical activity, and social support, to enhance distress tolerance.

From a clinical perspective, interventions should focus on strengthening distress tolerance and emotion regulation skills. Approaches such as Dialectical Behavior Therapy (DBT) and cognitive-behavioral interventions may be particularly useful, as they target both emotional regulation and maladaptive coping patterns. Clinicians should also consider both substance use and underlying psychological distress during assessment and treatment, as these processes appear closely intertwined.

At the institutional level, universities should adopt integrated and non-judgmental approaches to student mental health and substance use. Creating safe and confidential environments for disclosure, along with incorporating substance use screening into routine services, may improve early identification and support. Interventions can be tailored to students' lived experiences, including academic stress and social anxiety, and may benefit from combining mental health support with practical resources such as academic counseling.

From a public health perspective, prevention strategies should move beyond punitive or purely abstinence-based approaches. A harm reduction framework that acknowledges the perceived benefits of cannabis while providing accurate information about risks may be more effective. In addition, addressing stigma is critical, particularly in contexts such as Türkiye where substance use is highly stigmatized. Promoting non-stigmatizing communication and framing substance use as a health issue rather than a moral failing may facilitate help-seeking and engagement with support services.

### Limitations

While this study yields valuable insights, several limitations must be acknowledged to interpret the findings appropriately. First, the sample was drawn from university students in three major cities in Türkiye and was not randomly selected. We used a combination of convenience and snowball sampling for the survey, and then purposively sampled high-risk individuals for interviews. As such, our sample may not be fully representative of all Turkish university students. Those who volunteered for a study on cannabis likely include a higher proportion of frequent users and students open about their use. Students who abstain or who use but fear disclosure might be underrepresented. This sampling bias means the prevalence estimates and even the strength of associations observed might not generalize to more hidden users or to other regions (e.g., more conservative rural areas or different universities). Relatedly, because the qualitative phase deliberately focused on students with *elevated* cannabis problems or psychological distress, the themes identified may emphasize the more problematic end of the spectrum. Caution is needed in extending conclusions to casual or infrequent cannabis users—our study was exploratory and intentionally targeted those experiencing issues, to maximize informational richness, but that comes at the cost of generality.

Second, the study relied on self-report measures for both the survey (questionnaires) and the interviews. Self-report is inherently vulnerable to certain biases. Notably, substance use can be under-reported due to social desirability or fear of repercussions—a concern particularly salient in the Turkish context where cannabis is illegal and stigmatized. We assured participants of confidentiality, but some may still have minimized their use or related problems. Similarly, self-reported symptoms of anxiety or depression can be influenced by current mood and willingness to disclose. The cross-sectional survey design cannot confirm causality; as discussed, we cannot definitively say whether cannabis use led to higher depression, or depressed mood led to more cannabis use, or a third factor (like a difficult life event) caused both. The directionality of effects is likely bidirectional and dynamic, as suggested by both our qualitative narratives and prior longitudinal research showing mixed causal pathways. Our integrated model hypothesizes a feedback loop, but we must be careful to label it as a hypothesis supported by indirect evidence, not proven fact. Longitudinal data would be needed to verify that, for example, low distress tolerance at baseline predicts subsequent cannabis escalation and that using cannabis chronically then erodes distress tolerance further. Without such data, temporal inferences remain speculative.

Another limitation is that our quantitative measures, while well-validated (CUPIT for cannabis problems, BDI-II, BAI, DTS), provide a snapshot of recent symptoms and behaviors but might not capture all relevant dimensions. For instance, the Distress Tolerance Scale assesses perceived tolerance, but we did not include a behavioral measure of distress tolerance; some research suggests discrepancies can exist between what people *think* they can tolerate and how they behave when distressed. We also did not directly measure other potentially important variables, such as trauma history, personality factors (like impulsivity or sensation-seeking), or social support, which could influence both psychological distress and substance use. Our focus was on a specific set of emotion-related constructs, which might oversimplify the complex tapestry of factors in a student's life. The qualitative data did bring up some of these contextual factors (e.g., peer influences, academic pressure, COVID-19 pandemic stress), but our analysis centered on common themes and may not have explored every divergent case or minority perspective in depth. It's possible that some interviewees had unique motives (for example, purely recreational enjoyment or curiosity) that were less emphasized in our thematic analysis due to our focus on psychological experiences.

Another limitation of the study is that potential gender differences were not examined. Although the sample included both male and female participants, the study was not specifically designed or powered to conduct gender-based subgroup analyses. Prior research suggests that gender may influence substance use motives, emotional regulation strategies, and psychological outcomes. Future studies should therefore investigate gender-specific patterns in cannabis use and its psychological correlates to provide a more nuanced understanding of these processes.

Additionally, there is a possibility of retrospective bias in qualitative reporting. Participants may rationalize or interpret their cannabis use through the lens of coping because that narrative is culturally or personally acceptable. For example, a student might say they smoke to deal with anxiety, when in fact multiple motives are in play (including simply liking the high). We tried to probe and validate accounts, but the data are still subjective reports. Moreover, the qualitative interviews were conducted after the survey, meaning participants already knew the study was examining psychological factors—this may have unconsciously primed them to frame their stories in terms of stress, anxiety, etc. We attempted to mitigate this by using open-ended questions and letting themes emerge inductively, but the risk of confirmation bias (both in participants and researchers) cannot be entirely ruled out.

Finally, the study's context, while a strength in terms of breaking new ground in Türkiye, is also a limitation for generalizability beyond this context. Cultural norms around cannabis, help-seeking, and emotional expression vary widely. For instance, in countries where cannabis is legal and widely accepted, students might openly discuss different experiences (perhaps more social use and less guilt) compared to our Turkish sample. Therefore, some of the dynamics we observed (like pronounced stigma-related distress) might be less applicable elsewhere. Conversely, the low base-rate of cannabis users in Türkiye might mean those who do use are a more “distinct” group (e.g., possibly more risk-taking or connected to particular subcultures) than in places where half of students use. These nuances mean that replication in other cultural settings is necessary to test which aspects of our findings are universal vs. culture-specific.

### Directions for future research

Future research should adopt longitudinal designs to clarify the temporal and potentially bidirectional relationships between psychological distress and cannabis use. Experience sampling methods may further elucidate moment-to-moment associations between affect and use behavior. Expanding the range of examined variables—such as coping motives, personality traits, and social context—would provide a more comprehensive understanding of risk and protective factors. Additionally, intervention-focused studies targeting emotion regulation and distress tolerance are warranted to evaluate strategies for reducing maladaptive coping through substance use. Future research should also explore potential gender differences in cannabis use, coping motives, and psychological outcomes.

## Conclusion

In conclusion, this mixed-methods exploratory study offers a nuanced understanding of how cannabis use and psychological wellbeing are intertwined among university students in Türkiye. By integrating quantitative breadth with qualitative depth, we were able to identify not only that *higher cannabis involvement is associated with greater emotional distress and poorer distress tolerance*, but also explore the lived meanings and mechanisms behind this association. The findings coalesce into a coherent narrative: many students appear to be caught in a cycle where emotional pain and cannabis use reinforce one another. Cannabis is frequently used as a quick-acting tool for emotion regulation—a way to self-soothe anxiety, lift mood, or escape stress—which validates classic theories of self-medication and negative reinforcement in a real-world student context. However, this strategy, while effective in the short term, was reported to have insidious longer-term effects: it diminished students' confidence and ability to cope without the substance and contributed to new problems (academic decline, guilt, relationship strains) that ultimately feed back into greater psychological distress. Our study thereby highlights a *vicious circle* that has both theoretical significance and practical urgency.

Crucially, this research extends the conversation beyond Western settings, providing one of the first detailed looks at these dynamics in a non-Western (Turkish) student sample. In doing so, it sheds light on cultural factors—notably stigma and secrecy—that amplify the psychological burden of cannabis use in this context. Despite these contextual differences, the core patterns observed are strikingly consonant with international literature, suggesting that emerging adulthood is a period of vulnerability to substance-related coping across cultures. The contribution of this study lies in its holistic approach: we have not only documented statistical relationships but also given voice to student experiences, thereby generating a more empathic and contextually grounded understanding. These insights can inform tailored interventions on Turkish campuses and offer comparative data for global health discussions on young adult cannabis use.

In summary, *Cannabis Use and Psychological Experiences Among University Students in Türkiye* provides evidence that problematic cannabis use among students is rarely just about seeking pleasure or peer influence—it is often symptomatic of underlying emotional struggles and a limited capacity to tolerate distress. Addressing this issue will require integrated strategies that treat the “whole student,” equipping them with healthier ways to manage the challenges of emerging adulthood. We hope that this study serves as a foundation for future research and action: by illuminating the feedback loop between distress and cannabis use, we underscore the need for compassionate, comprehensive approaches to break that cycle and support the wellbeing and success of university students in Türkiye and beyond.

## Data Availability

The original contributions presented in the study are included in the article/supplementary material, further inquiries can be directed to the corresponding author.
